# AFM-Based Characterization Method of Capacitive MEMS Pressure Sensors for Cardiological Applications

**DOI:** 10.3390/mi9070342

**Published:** 2018-07-06

**Authors:** Jose Angel Miguel, Yolanda Lechuga, Mar Martinez

**Affiliations:** Group of Microelectronics Engineering, Department of Electronics Technology, Systems Engineering and Automation, University of Cantabria, Santander 39005, Spain; jamd@teisa.unican.es (J.A.M.); martinez@teisa.unican.es (M.M.)

**Keywords:** micro-electro-mechanical systems (MEMS) sensors, MEMS modelling, capacitive pressure sensor, MEMS characterization, atomic force microscope, stent

## Abstract

Current CMOS-micro-electro-mechanical systems (MEMS) fabrication technologies permit cardiological implantable devices with sensing capabilities, such as the iStents, to be developed in such a way that MEMS sensors can be monolithically integrated together with a powering/transmitting CMOS circuitry. This system on chip fabrication allows the devices to meet the crucial requirements of accuracy, reliability, low-power, and reduced size that any life-sustaining medical application imposes. In this regard, the characterization of stand-alone prototype sensors in an efficient but affordable way to verify sensor performance and to better recognize further areas of improvement is highly advisable. This work proposes a novel characterization method based on an atomic force microscope (AFM) in contact mode that permits to calculate the maximum deflection of the flexible top plate of a capacitive MEMS pressure sensor without coating, under a concentrated load applied to its center. The experimental measurements obtained with this method have allowed to verify the bending behavior of the sensor as predicted by simulation of analytical and finite element (FE) models. This validation process has been carried out on two sensor prototypes with circular and square geometries that were designed using a computer-aided design tool specially-developed for capacitive MEMS pressure sensors.

## 1. Introduction

Cardiovascular diseases are the predominant cause of mortality worldwide [1,2]. The 2013 Global Burden of Disease study estimated that they were responsible for more than double the deaths than that caused by cancer. In the European Union alone, they accounted for almost 40% of all deaths in 2013, and ischemic heart diseases (IHD) alone were responsible for more than 35% of deaths. These conditions are caused by the accumulation of fatty deposits lining the inner wall of a coronary artery, restricting blood flow to the heart.

Patients diagnosed with IHD are commonly subjected to a surgical procedure called percutaneous coronary intervention (PCI), in which the regular blood-flow in a clogged vessel is usually restored and maintained by the implantation of a biocompatible mesh tube or Stent. Nevertheless, neo-intimal tissue growth inside the stent (in-Stent restenosis, ISR) stands out as its major drawback, jeopardizing patients’ life and forcing, in many cases, the repetition of the procedure. Current tracking methods for ISR are expensive and time-consuming as they require complex equipment, specialized medical staff, and even patient’s hospitalization. Thus, the proposal of intelligent stents (iStent) endowed with blood-flow and/or pressure sensing capabilities represents a potential economic solution that, nonetheless, must be reliable, efficient, compact, low-power, and less expensive than its counterparts to be considered as an actual alternative.

In this sense, the arising of an affordable fabrication technology of micro-electro-mechanical systems (MEMS), which combine an integrated circuit (IC) with mechanical parts, permits these stents with tracking capabilities to meet the aforementioned requirements.

Among the different options for MEMS pressure sensors [3,4], this work is based on a capacitive approach in which sensors are conceived as parallel-plate capacitors with a fixed and a flexible plate that bends with increasing pressure. The IC will measure and transmit the equivalent capacitance of the sensor that will reflect the decrease in the dielectric gap by deflection of the upper plate and, thus the applied pressure.

However, before undertaking the task of developing a monolithic heterogeneous IC that includes the electronic circuitry and the MEMS sensor on the same substrate, it is advisable to characterize stand-alone prototype sensors in an efficient but affordable way to better recognize further areas of improvement. On the one hand, these prototypes are to be fabricated using MEMS technology that allows for the development of several sensor types at a low cost. On the other hand, a fast characterization method should have direct access to the mechanical structure of the sensor in order to directly measure the deflection of the upper plate and, thus to characterize its detection capability and sensitivity.

The simplest approach to MEMS pressure sensor’s experimental characterization implies the design and fabrication of a measurements setup, in order to expose the device under test (DUT) to an accurately controlled pressure [5]. However, this characterization methodology is only suitable for fully sealed MEMS sensors, with their inner gap isolated from the outer environment. For the case of biosensors similar to the ones described in this paper, a biocompatible coating is required to both seal the structure and guarantee its reliable operation once implanted. However, a characterization of the uncoated DUT provides significant advantages: First, the evaluation of the sensor sensitivity loss and offset change as a result of the coating [5]. Second, the acquisition of performance data from the bulk sensor highlights the need for further design adjustments, prior to its monolithic integration. Third, the possibility of double-checking the sensor response (before and after coating) helps to improve its reliability, being a critical aspect for the development of implantable electronic devices. Finally, non-requiring pressure-controlled environments for testing setups provides a significant cost-saving solution for the sensor characterization problem [5].

**Figure 1:**
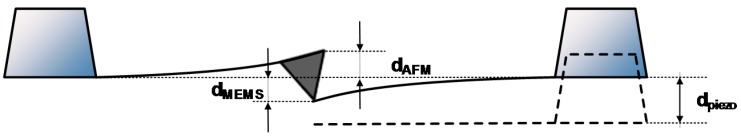
Concept of sensor characterization by the atomic force microscope (AFM) operation in contact mode (not to scale). MEMS—micro-electro-mechanical systems.

Hence, the performance parameters of non-coated MEMS pressure biosensors cannot be collected from pressure-based measurements, so another method based on an alternative excitation signal must be used. It must be highlighted that the sensor response to this substitute stimulus has to be formerly modelled analytically and/or numerically, in order to allow the validation of the collected experimental data. In this sense, a novel approach for the characterization of uncoated MEMS pressure biosensors, based on an atomic force microscope operating in contact mode, is proposed in this paper. The atomic force microscope (AFM) is a relatively common laboratory instrument, and this methodology takes advantage of its highly sensitive optical lever detection system and ability to position a probe with nanometer precision, in order to apply a known concentrated load directly on the center of the upper and flexible plate of the sensor. By this way, the deflection of the sensor can be calculated according to the concept shown in Figure 1.

In this work two prototypes of capacitive MEMS pressure sensors, with circular and square shapes, have been fabricated using PolyMUMPS technology. This MEMS fabrication technology has been selected for being mature and generalist, in the sense that it can accommodate different MEMS structures and also allows sensor prototyping without coating.

The bending of the AFM probe can be calculated by Hook’s law:

(1) $$F=k_{AFM}\cdot \Delta z$$

where F is the force applied by the AFM probe, $k_{AFM}$ is its spring constant, and Δz is the relative displacement of the probe from the equilibrium position where the applied force is zero.

Assuming that once the equilibrium has been reached, the forces acting on the AFM probe and the sensor under test are the same. Thus, it can be stated that $k_{AFM}d_{AFM}=k_{MEMS}d_{MEMS}$, where $d_{AFM}$ and $d_{MEMS}$ are the AFM and sensor deflections, respectively. Considering an AFM piezo vertical displacement $d_{piezo}$ during the measurement required to move the sample position, it is noticeable that $d_{piezo}$ combines both the AFM probe and MEMS sensor displacements, as shown in Figure 1. Thus, the resulting sensor displacement $d_{MEMS}$ can be calculated as follows [6,7,8,9]:

(2) $$d_{MEMS}=d_{piezo}-d_{AFM}$$

In Patil et al. [6], an AFM in contact mode is used for the characterization and calibration of a piezoresistive pressure sensor designed for tactile sensing applications. In this paper, the probe-tip was modified by attaching a spherical soda-lime glass particle to its end so as to increase the contact area and simulate a uniform pressure application.

The goal of Alici et al. [7] is to characterize the stiffness of microfabricated cantilevers by utilizing an AFM to develop a static deflection measurement method. More specifically, the AFM is used to apply a known load at the end of a polymer microactuator so as to determine the spring constant from the resulting displacement and a reference calibration method. In this sense, the paper emphasizes the need of a previous calibration step to determine the spring constant of the AFM cantilever.

On the other hand, Rollier et al. [8] take advantage of the AFM features to obtain measurements of force and resonant frequency so that the value of the tensile residual stress of silicon nitride membranes could be extracted. Thus, the objective of this works lies in developing a non-destructive method that permits to improve the low stress silicon nitride deposition process and to optimize released membrane fabrication, not to characterize the behavior of the membrane itself as part of a sensing device.

To summarize, and as stated in Pustan et al. [9], AFMs can be utilized to evaluate the mechanical properties of micro/nanoscale structures and nanomaterials used in MEMS and NanoElectroMechanical Systems (NEMS). More specifically, this work concludes that the dependency between an acting force and the sample deflection is determined by the AFM static mode, and from that data, the stiffness of the microcantilever can be computed. Moreover, the modulus of elasticity of the material is derived by nanoidentation. Besides, AFM-based measurements have been reported to be of great use to characterize pressure sensors, for example, of the piezoresistive type [6]. However, no literature has been found that treated the problem of both analytically and Finite Element (FE) modelling the deflection versus force behavior of non-coated capacitive MEMS pressure biosensors, together with their experimental characterizing using an AFM in contact mode, used to apply a force that permits to estimate the corresponding deflection of the top plate and, thus its sensitivity.

The complete design and characterization process of the MEMS capacitive sensor explained in this work also includes an extensive modelling stage, in which analytical and finite element models have been developed, and are described in Section 2 of this document. This section also includes a description of how a computer-aided design tool specifically developed for capacitive MEMS pressure sensors would guide the designer through the main steps of the process, from the specifications and technology, to the FE model and Cadence layout of the sensor, ready to be sent to the manufacturer. Finally, Section 3 presents the experimental measurements obtained with the AFM compared with the simulation data provided by the analytical and FE models. The results are further discussed in the final section to evaluate the efficiency of the AFM-based characterization method, as well as to propose areas of potential improvement.

## 2. Materials and Methods

### 2.1. Capacitive MEMS Pressure Sensor Modelling

**Figure 2:**
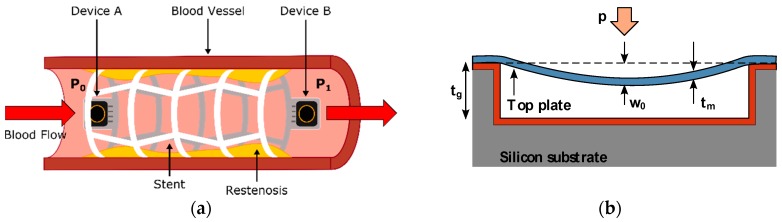
Building parts of a passive pressure sensing iStent. (**a**) Lateral view of the iStent implanted inside a blood vessel; (**b**) cross-sectional view of a MEMS capacitive pressure sensor.

The simplest implementation of a pressure monitoring-based iStent, as show in Figure 2a, can be built by attaching one or several MEMS capacitive pressure sensors to the longitudinal ends of a commercially available stent. The device functionality is based on the blood pressure detection performed by the sensors, reflecting on proportional changes in their equivalent capacitance [3,4]. Thus, MEMS sensors act as pressure-dependent capacitances, which, attached to the coil-like stent structure, form an LC-tank whose resonant frequency is modulated by the pressure inside the vessel. An external handheld device is required to perform the wireless communication with the iStent, via inductive coupling techniques, when placed close enough to the implant location.

The topology of a capacitive MEMS pressure sensor, as described in Figure 2b, comprises a fully clamped suspended top plate with thickness $t_{m}$, separated a distance of $t_{g}$ from a backplate fixed to the substrate. As can be noticed, this topology resembles the traditional parallel-plate capacitor build, so its nominal capacitance is ruled by Equation (3):

(3) $$C_{0}=\varepsilon_{r}\varepsilon_{0}\frac{A}{t_{g}}$$

where $\varepsilon_{r}$ is the relative permittivity of the medium between the plates, $\varepsilon_{0}$ is the dielectric permittivity of vacuum, and A and $t_{g}$ are the overlapping area and gap distance between the plates, respectively.

Once a sufficient load p is applied to the sensor, the suspended plate is forced to bend towards the backplate, in such a way that their separation is reduced and the equivalent capacitance $C_{S}$ is increased. Hence, the resulting load-dependent capacitance can be analytically modeled by Equation (4), where parameter w(x,y,p) refers to the local top-plate deflection.

(4) $$C_{S}=\int \int_{A}\frac{\varepsilon_{r}\varepsilon_{0}dxdy}{t_{g}-w(x,y,p)}$$

#### 2.1.1. Square Sensor Modelling

As extensively reported in Mechanics books and scientific papers, the governing differential equation for the deflection of a thin plate in cartesian coordinates can be expressed as follows [10,11]:

(5) $$\frac{\partial^{4}w}{\partial x^{4}}+2\frac{\partial^{4}w}{\partial x^{2}\partial y^{2}}+\frac{\partial^{4}w}{\partial y^{4}}=\frac{p}{D}$$

where w(x,y) is the deflection of the square plate at any place, p is a distributed load applied to the upper surface of the plate, and D is referred as the flexural rigidity of the plate and can be defined as follows:

(6) $$D=\frac{Et_{m}^{3}}{12(1-v^{2})}$$

with E and v being the modulus of elasticity and Poisson’s ratio of the plate material, respectively.

**Figure 3:**
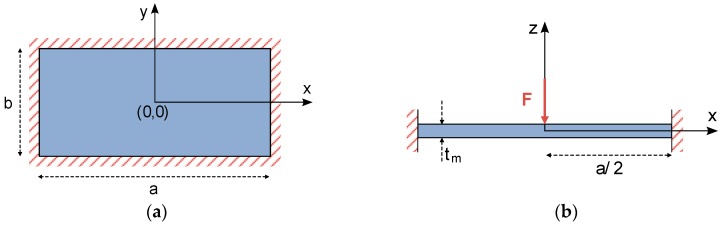
Schematic representation of a rectangular plate under a central concentrated load. (**a**) Top view of the plate; (**b**) cross-sectional view of the plate.

In the case of a fully-clamped square plate with side lengths b=a and thickness $t_{m}$, such as the one showed in Figure 3a, the following set of constraints describe the plate bending behavior and can be used to solve the differential equation noted in (5).

(7) $$w(x=\pm \frac{a}{2},y)=0$$

(8) $$w(x,y=\pm \frac{b}{2})=0\;$$

(9) $$\frac{\partial w}{\partial x}(x=\pm \frac{a}{2},y)=0\;$$

(10) $$\frac{\partial w}{\partial x}(x,y=\pm \frac{b}{2})=0\;$$

Once a single concentrated load is applied to the center of the square plate (Figure 3b), its deflection equation can be calculated by combining the solutions for three independent problems [10,11,12]. First, $w_{1}$ is the bending solution for a simply supported rectangular plate under a concentrated load located at its center, and can be expressed as follows:

(11) $$\begin{array}{l} w_{1}(x,y)=\frac{pa^{2}}{2\pi^{3}D}\sum_{m=1,3,5,\ldots }\frac{1}{m^{3}}\cos\frac{m\pi x}{a}[(\tanh a_{m}-\frac{a_{m}}{\cosh^{2}a_{m}})\cos\frac{m\pi y}{a}-\sinh\frac{m\pi y}{a}\\ -\tanh a_{m}\frac{m\pi y}{a}\sinh\frac{m\pi y}{a}+\frac{m\pi y}{a}\cosh\frac{m\pi y}{a}] \end{array}$$

where the geometry parameters $a_{m}$ and $\beta_{m}$, defined as $a_{m}=m\pi b/2a$ and $\beta_{m}=m\pi a/2b$, can be reduced to mπ/2 for the case of a square plate with sides a=b.

Additionally, $w_{2}$ and $w_{3}$ are the bending solutions for a simply supported plate with distributed bending moments applied along the edges y=±b/2 and x=±a/2, respectively. The applied edge moments are calculated to guarantee a slope at the boundaries equal to zero, as imposed by constraints (9) and (10).

Hence, each aforementioned solution can be defined as follows:

(12) $$\begin{array}{l} w_{2}(x,y)=-\frac{a^{2}}{2\pi^{2}D}\sum_{m=1,3,5,\ldots }a_{m}\frac{{\left(-1\right)}^{\frac{m-1}{2}}}{m^{2}\cosh a_{m}}\cos\frac{m\pi x}{a}[\frac{m\pi y}{a}\sinh\frac{m\pi y}{a}\\ -a_{m}\tanh a_{m}\cosh\frac{m\pi y}{a}] \end{array}$$

and

(13) $$\begin{array}{l} w_{3}(x,y)=-\frac{b^{2}}{2\pi^{2}D}\sum_{m=1,3,5,\ldots }B_{m}\frac{{\left(-1\right)}^{\frac{m-1}{2}}}{m^{2}\cosh\beta_{m}}\cos\frac{m\pi y}{b}[\frac{m\pi x}{b}\sinh\frac{m\pi x}{b}\\ -\beta_{m}\tanh\beta_{m}\cosh\frac{m\pi x}{b}] \end{array}$$

The coefficients $A_{m}$ and $B_{m}$ can be determined from the fully-clamped plate constraints (9) and (10), or the condition of cancelling the slope at the plate boundaries. Hence, the most significant $A_{m}$ and $B_{m}$ values for a square plate, given in Table 1, can be numerically calculated by successive approximations.

To conclude, the combination of Equations (11)–(13) leads to the final bending solution:

(14) $$w(x,y)=w_{1}(x,y)+w_{2}(x,y)+w_{3}(x,y)$$

#### 2.1.2. Circular Sensor Modelling

**Figure 4:**
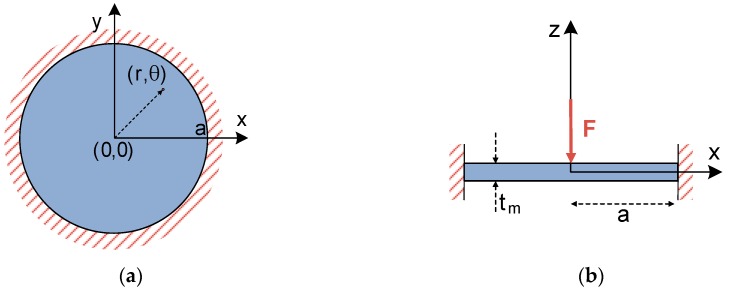
Schematic representation of a circular plate under a central concentrated load. (**a**) Top view of the plate; (**b**) cross-sectional view of the plate.

Similar to the case of a square plate, the analytical bending solution for a fully-clamped circular thin plate under a concentrated central force has been widely studied in the literature [10,11]. Hence, differential Equation (5) determining plate deflection can be rearranged in polar coordinates [10,11], and easily applied to a circular plate, such as the one included in Figure 4.

(15) $$\nabla_{r}^{4}w\equiv (\frac{\partial^{2}}{\partial r^{2}}+\frac{1}{r}\frac{\partial }{\partial r}+\frac{1}{r^{2}}\frac{\partial^{2}}{\partial \theta^{2}})(\frac{\partial^{2}w}{\partial r^{2}}+\frac{1}{r}\frac{\partial w}{\partial r}+\frac{1}{r^{2}}\frac{\partial^{2}w}{\partial \theta^{2}})=\frac{p}{D}$$

with w(r,θ) being the bending of the circular plate in polar coordinates, and p the distributed load applied to its upper surface. Moreover, the boundary constraints for the plate center and perimeter (r=a) can be expressed as follows:

(16) w(r=a,θ)=0

and

(17) $$\frac{\partial w}{\partial r}(r=a,\theta )=0$$

Unlike the previous case, the bending solution for the fully clamped circular sensor can be obtained in a purely analytical way. By differentiating the bending solution for a simply supported plate and forcing the slope to nullify at the boundary, as imposed by (17), it is possible to calculate the bending moments along the plate edges. The final solution can be calculated by adding the deflection produced by the moments along the edges to the initial simply supported bending equation, obtaining the following expression:

(18) $$w(r,\theta )=\frac{pr^{2}}{8\pi D}\ln\frac{r}{a}+\frac{p}{16\pi D}(a^{2}-r^{2})$$

#### 2.1.3. Modelling Results Comparison

In order to characterize the accuracy of the bending solutions presented in the previous subsections, the responses provided by analytical equations have been compared with the ones obtained from equivalent finite elements (FE) models; so that the full scale error (FSE) between both modelling approaches could be calculated.

Two capacitive polysilicon-based (E=169 GPa; v=0.22) MEMS pressure sensors, circular and square-shaped, have been designed. A self-developed computed aided design (CAD) tool described in Section 2.2 has been used for this purpose. An initial design constraint has been imposed to both sensors, forcing their dimensioning to reach an equal nominal capacitance of $C_{0}=0.6\;\mathrm{pF}$.

**Figure 5:**
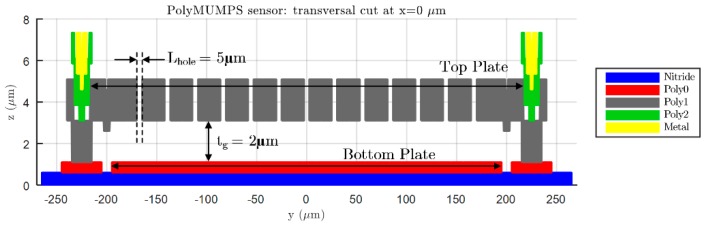
View of the transversal cut at x=0 μm for a PolyMUMPS squared sensor, based on a top bottom plate configuration with sides $a_{bottom}=390\;\mu m$ and $a_{top}=410\;\mu m$, respectively.

**Figure 6:**
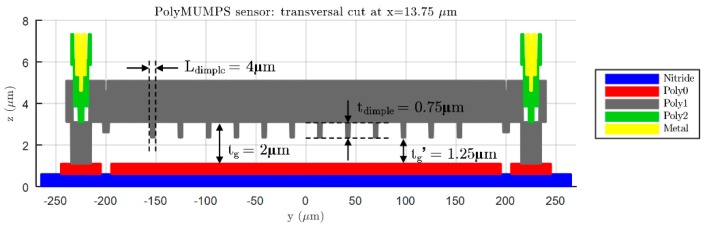
View of the transversal cut at x=13.75 μm for a PolyMUMPS squared sensor, based on a top bottom plate configuration with sides $a_{bottom}=390\;\mu m$ and $a_{top}=410\;\mu m$, respectively.

As can be seen in Table 2 and Table 3, two different FE models with variable complexity have been developed in ANSYS, in order to perform displacement versus force simulations and compare the resulting bending data with the response anticipated by the analytical expressions (14) and (18). A first FE model, referred as “simple”, consists of a flat square or circular plate fully clamped along its edges. This model requires low computational time in ANSYS to achieve a complete bending characterization of the sensor, because of its relatively low complexity. On the other hand, the second FE model, named “complex”, presents the exact same topology as the prototype sensor fabricated in PolyMUMPS technology. In this case, hole and dimple elements have been added because of the requirement imposed by the manufacturer for diaphragms larger than 30 μm×30 μm [13]. The former elements are square-shaped through-holes with a side length of $L_{hole}=5\;\mu m$, required to provide shorter release etch paths for the removal of the sacrificial layer, as can be seen in Figure 5. The latter elements are polysilicon elements of $t_{dimple}=0.75\;\mu m$ height, placed under the suspended diaphragm in order to limit the contact surface and reduce the plate stiction occurrence [13], as showcased in Figure 6. Moreover, a lateral opening of side $L_{open}=50\;\mu m$ has been added to the top plate anchoring structure, in order to facilitate the bottom plate electrical routing to the sensor bonding PADS. As expected, the greater complexity of this later model provokes a significant increase of the simulation time required to characterize its bending versus force behavior in ANSYS. However, the “complex” FE model produces more accurate results, showcasing an increased sensor sensitivity to the applied load, caused by the presence of hole cavities on the top plate; as well as a realistic contact point between the plates obtained under the presence of dimple elements, which reduce the plates gap in $t_{dimple}=0.75\;\mu m$ [13].

**Figure 7:**
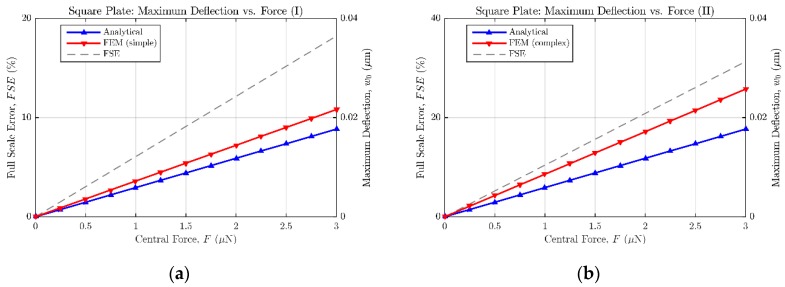
Maximum deflection versus central force for a fully clamped square plate. (**a**) Analytical model and finite element (FE) simple model results; (**b**) analytical model and FE complex model results.

**Figure 8:**
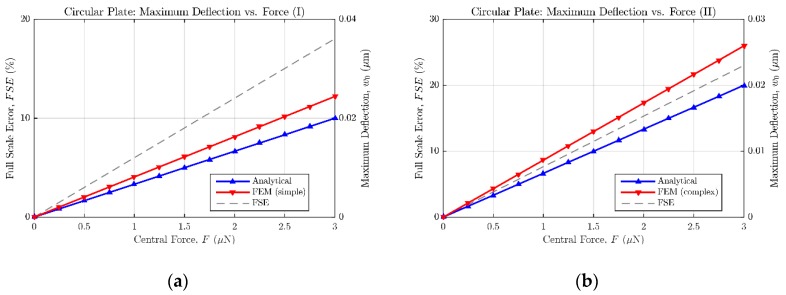
Maximum deflection versus central force for a fully clamped circular plate. (**a**) Analytical model and FE simple model results; (**b**) analytical model and FE complex model results.

As a result of the aforementioned modelling, Figure 7 and Figure 8 show the maximum bending estimation against the concentrated force applied to the sensors described in Table 2 and Table 3, respectively. Both the analytical and FE responses are presented in each figure to ease the comparison between models. Additionally, the full scale error value is provided to quantify the mismatch between both models. According to the collected results, it can be stated that the analytical expressions (14) and (18) tend to underestimate the bending of the plates, when compared with the results of the FE models. However, because of their easy translation to the mathematical software (Matlab) and their fast evaluation, the analytical approach can still be considered an accurate-enough method to estimate the sensor behavior in an efficient way, previous to the FE model implementation in ANSYS. Alternative numerical approximation methods can be used to enhance the achieved exactitude, at the expense of immediacy, increased complexity, and higher computing requirements.

### 2.2. CardioMEMS Design Tool

**Figure 9:**
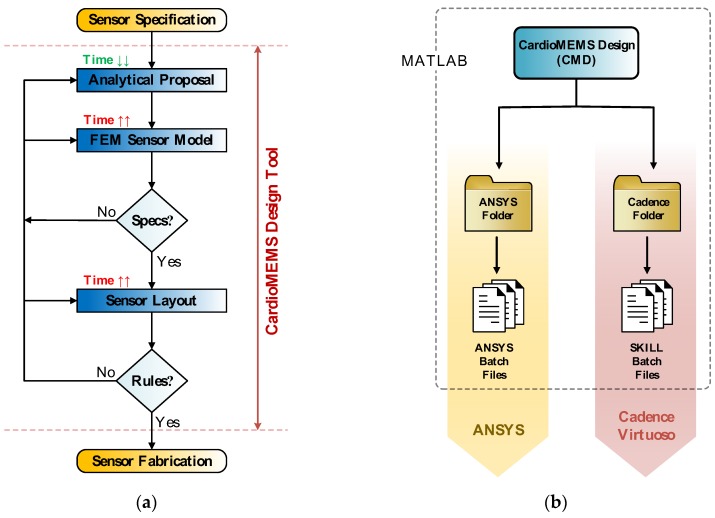
CardioMEMS Design (CMD) functionalities overview. (**a**) MEMS sensor design flow, highlighting those stages covered by CMD; (**b**) CMD output folders generated to export the designed sensor to ANSYS and Cadence Virtuoso.

MEMS sensor design flow, as shown in Figure 9a, comprises three main stages. First, a rough sensor design proposal is obtained by the evaluation of a set of analytical expressions, while satisfying the initial design constraints. Second, the sensor realistic 3D structure is implemented in a modelling software for FE analysis; in order to perform the necessary simulations to achieve an accurate characterization of the device behavior. Finally, the third stage involves the sensor layout description in the corresponding technology layers being sent to the manufacturer. CardioMEMS Design (CMD) is a Matlab-based computer aided design (CAD) tool developed with the aim of automating the aforementioned design steps, as well as providing a friendly interface to guide the user through the sensor design process [14,15]. Thus, as exposed in Figure 9b, CMD provides as outputs the set of files required to export the designed sensor to ANSYS and Cadence Virtuoso working environments, to perform different FE analyses and build the sensor layer description, respectively.

**Figure 10:**
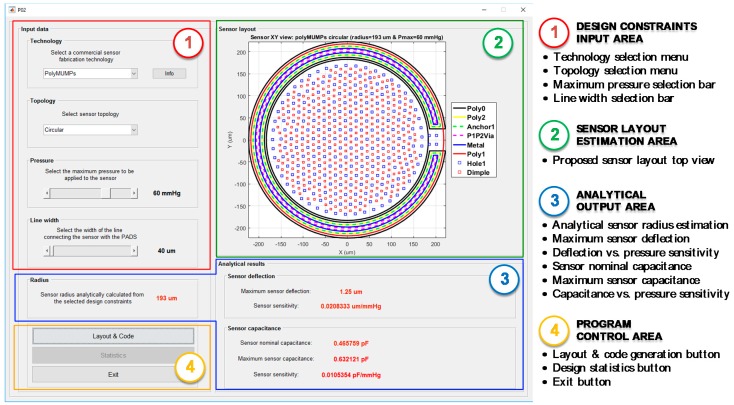
CardioMEMS Design (CMD) main screen, with its working areas and main functionalities displayed.

The use of CMD to perform the complete design of a capacitive MEMS pressure sensor for ISR-monitoring iStents is described next, in order to present its functionalities and features. First, as can be seen in Figure 10, CMD requires the definition of various input parameters to provide an initial sensor design, including the selection of a fabrication technology included in the database, the preferred sensor topology, the maximum pressure to be borne by the transducer, and the desired width of the line used to physically connect the sensor to the bonding PADs.

In the case of our prototype sensors, the selected MEMS technology has been PolyMUMPS by MEMSCAP, a mature and reliable surface-micromachining fabrication process, developed to accommodate a wide variety of MEMS structures. PolyMUMPS uses eight masks to define the topology of seven physical layers: three polysilicon layers, a metal (Au) layer, and two phospho-silicate glass (PSG) sacrificial layers [13]. It is important to keep in mind that the information about any new fabrication process must be added to the CMD database to allow for the use of that particular technology. Second, any MEMS pressure sensors intended to be used for mild ISR detection in a distal ramification of the pulmonary artery must face pressures in the range of P=[0,60] mmHg [16], while presenting the maximum achievable sensitivity to pressure changes. Thus, a circular sensor topology has been chosen initially, because of its higher sensitivity compared with a squared-shaped sensor of the same area [14]. Finally, a 40 µm wide Polysilicon line has been selected to physically connect the sensor to the bonding PADs. Table 4 includes a summary of the input parameters introduced in CMD’s Design Constraints Area.

Once the input parameters have been defined, CMD determines the optimum sensor radius a by evaluation of the analytical equations for the deflection of a circular or square plate under a uniformly applied pressure. Internally, the program considers that the peak deflection, the contact between the suspended top plate and the fixed back plate, is achieved when the maximum pressure is applied to the sensor, thus imposing an additional constraint of $w_{0}=t_{g}$. As can be seen in Figure 10, the main performance parameters for the proposed sensor are displayed in the analytical output area of the program, including the sensor radius (a), its maximum deflection ($w_{0}$) and deflection versus pressure sensitivity ($S_{P}^{w_{0}}$), together with its nominal ($C_{0}$) and maximum capacitances ($C_{MAX}$) and the capacitance versus pressure sensitivity ($S_{P}^{C_{S}}$). Because of the relative simplicity of the analytical models used, every time one of the input parameters is modified, the output parameters are quickly recalculated and shown to the user. This allow the designer to evaluate different sensor technologies, topologies, and pressure ranges, getting an initial estimation of their performance in an efficient and agile way.

The *Layout & Code* bottom in the CMD main screen becomes active right after the initial design proposal has been presented to the user. By clicking on it, CMD internally performs a design adjustment; now taking into account the set of design rules provided by the technology manufacturer. Additionally, CMD considers the functional limitations from both ANSYS and Cadence Virtuoso while performing the design rearrangement, in order to guarantee its compatibility with both environments.

In the case of PolyMUMPS sensors, the optimum location for both holes and dimples is selected by CMD through the evaluation of a geometric distribution algorithm, based on Delaunay triangulation theory [17]. This algorithm minimizes the number of elements added to the structure, while guarantying the fulfillment of the design rules imposed by the technology. The rearranged sensor design is presented to the user in the sensor Layout Estimation Area, providing a top view of the layout displaying all the required technology layers for its fabrication, as shown in Figure 10.

Additionally, CMD saves relevant statistical information about the layers geometry. This information is accessible by the *Statistics* button in the program control area, as indicated in Figure 10, only after the *Layout & Code* operation has concluded.

**Figure 11:**
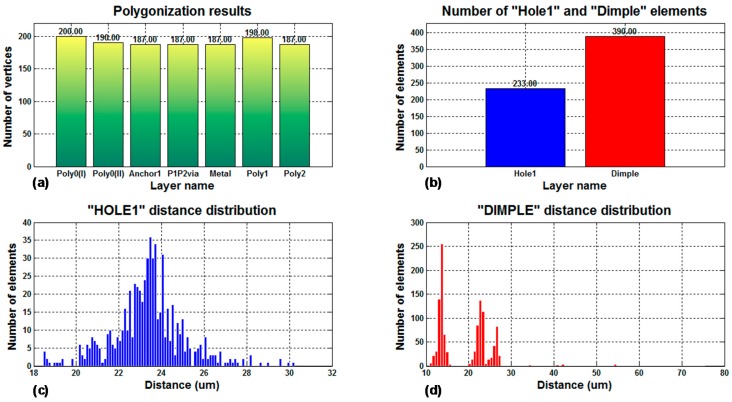
Sensor statistics provided by CMD, including (**a**) number of vertices for each fabrication technology layer; (**b**) number of “hole” and “dimple” elements included in the sensor structure; (**c**) distance distribution between “hole” elements; and (**d**) distance distribution between “dimple” elements.

Figure 11 includes a summary of the statistical data analyzed by the program for a PolyMUMPS-based sensor design. More concretely, Figure 11a provides the number of vertices used to polygonise the sensor defining layouts, which is a Cadence Virtuoso limiting operation factor. As can be noted, CMD fixes the number of vertices to a maximum value of 200 per layer, so Cadence Virtuoso constraints are not infringed. Moreover, CMD takes into account the spacing limitations between hole and dimple elements, defined in the provided PolyMUMPS documentation as $d_{hole}=[3,30]\;\mu m$ and $d_{dimple}\leq 3\;\mu m$, respectively [13]. The distance distribution for each type of element is displayed in Figure 11c,d; while Figure 11b indicates the total number of elements added to the basic sensor structure. This set of data is provided to the designer in order to quickly check that the design restrictions established by both the manufacturer and the software suites are thoroughly satisfied.

As previously mentioned, and illustrated in Figure 9b, one of the main goals of CMD is to facilitate the sensor design translation to ANSYS and Cadence Virtuoso, so that the final stages of the design flow can be completed. Hence, after each design completion, CMD generates two output folders, denoted as *codeAnsys* and *codeCadence*, comprising a series of files suitable to automatically export the sensor design to those platforms.

The *codeAnsys* folder contains seven files, with three of them being auto-executable files, while the remaining four files have an auxiliary purpose. Those three main batch code files, once individually loaded in ANSYS, build the 3D solid model of the sensor, optimize its FE meshing, and perform different simulations to completely characterize the device behavior. For instance, one of the files configures the program solver to calculate the sensor deflection and equivalent capacitance for a uniformly applied pressure in the intended operation range of $P=[0,P_{\mathrm{MAX}}]\;\mathrm{mmHg}$ Similarly, a second main file sets the solver to evaluate the sensor deflection and capacitance under the presence of a central force load (F), similar to the one that can be applied by an AFM microscope operating in contact mode [6,7,8,9]; and described in detail in Section 2.1. Finally, the last main code file arranges a modal analysis to determine the natural frequencies of the structure.

**Figure 12:**
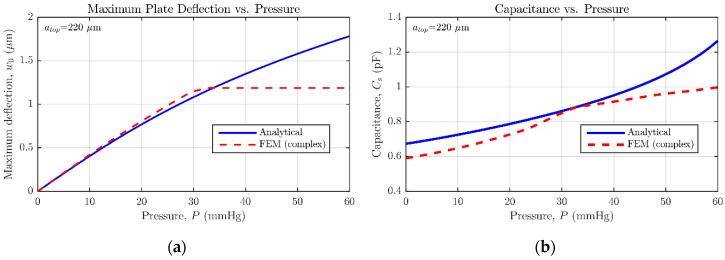
Comparison between the analytical and FEM models for circular MEMS pressure sensor with top plate radius of $a_{top}=220\;\mu m$. (**a**) center deflection versus pressure response; (**b**) capacitance versus pressure response.

In Figure 12, the central deflection and capacitance versus pressure simulation results are summed up. As can be observed, the simulation results given by ANSYS diverge slightly from those anticipated by the set of analytical expressions [14]. It must be acknowledged by the user that CMD proposes an initial rough sensor geometry based on the evaluation of analytical/numerical equations, because of their high computational efficiency. On the other hand, the CMD-rearranged design built in ANSYS presents higher complexity, mainly due to the addition of hole and dimple elements, responsible for reducing the stiffness of the movable plate [14] and limiting the effective gap distance to $t_{g}^{\prime }=t_{g}-t_{dimple}=1.25\;\mu m$, respectively. Furthermore, as detailed in Figure 5 and Figure 6, the realistic sensor model requires a moderately smaller backplate compared with the suspended one, which negatively affects the sensor nominal capacitance (Figure 12b). It can be perceived how the increased complexity of the structure contributes to locating the contact point in the range of measurable pressures; meaning that the initial sensor proposal underestimates the device sensitivity. However, for some applications, it can be desired to force the sensor operation entirely in contact mode, so a lineal capacitance response can be achieved [18]. The designer can take advantage of this behavior, being able to get exponential or linear capacitance versus pressure responses just by properly selecting the maximum detectable pressure at the beginning of the design flow.

**Figure 13:**
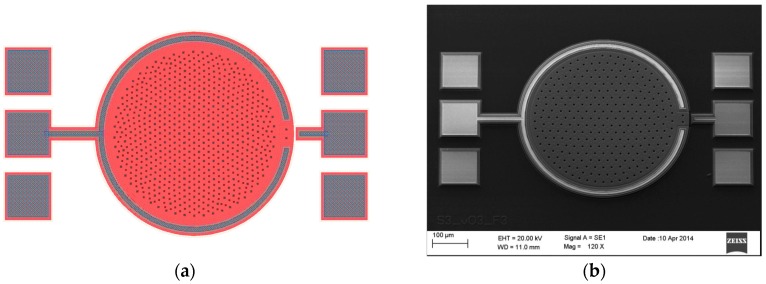
Final design stages for a circular MEMS pressure sensor with top plate radius of $a_{top}=220\;\mu m$. (**a**) Cadence Virtuoso layout view, generated through the SKILL batch files provided by CMD; (**b**) scanning Electron Microscope (SEM) picture of the fabricated sensor.

The *codeCadence* folder contains a unique auto executable SKILL batch file, supported by eighteen auxiliary files used to individually define the geometry of each fabrication layer. After being loaded in Cadence Virtuoso, the main batch file conducts the drawing of the necessary layers to create an adequate GDSII stream format file to be sent to the manufacturer. Figure 13a shows the layout view of the prototype sensor automatically built in Candence Virtuoso’s Layout Suite; where the bonding PADs are the only elements manually added by the designer. Besides, for comparison purposes, Figure 13b presents a scanning electron microscope (SEM) image of the fabricated sensor, defined by the layout in Figure 13a.

## 3. Results

**Figure 14:**
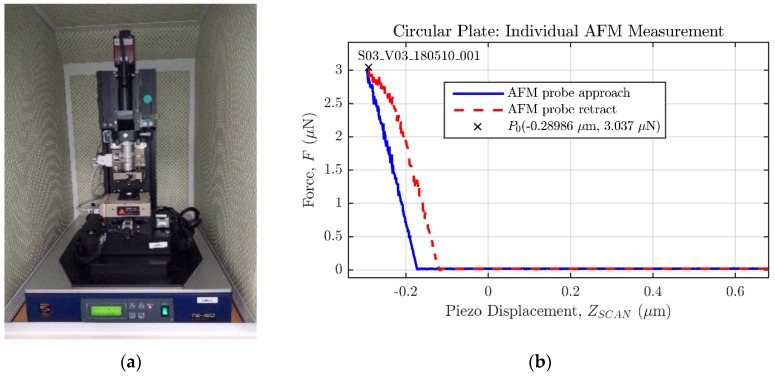
Experimental characterization equipment and sample measurement. (**a**) AFM model XE-100 by Park Systems; (**b**) sample contact mode measurement performed on a prototype circular MEMS sensor.

The deflection versus force response of two prototype PolyMUMPS square and circular pressure sensors, referred to as *S02_V03* and *S03_V03*, respectively, has been experimentally characterized using the atomic force micorscope model XE-100 by Park Systems (Figure 14a). These measurements were developed at the Centro Tecnológico de Componentes, CTC (Technological Centre of Components) in Santander, where the AFM machine is located.

The microscope mounts a probe PPP-NCHR with a spring constant of nominal value $k_{AFM}=42\;N/m$, and a tolerance range of [10,130] N/m, as specified by the manufacturer. The AFM has been configured to apply a maximum force of $F_{MAX}=3\;\mu N$ to the sensor plate, in order to obtain a force (F) against piezo displacement ($Z_{SCAN}$) curve similar to the one displayed in Figure 14b. Both sensors have been measured a total of 20 times, and during each individual measurement, the piezo displacement is varied in 4096 steps; so the resulting data set can be considered statistically significant.

**Figure 15:**
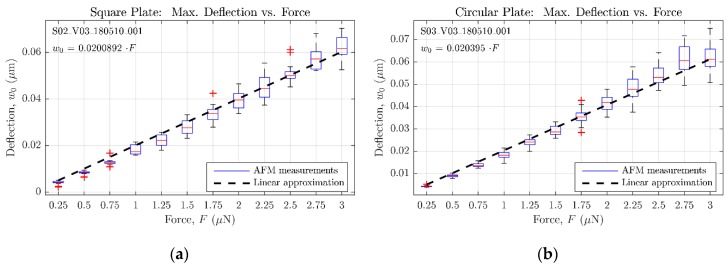
Experimental measurements of the sensor deflection versus a concentrated central force, assuming a AFM cantilever spring constant of $k_{AFM}=42\;N/m$. (**a**) For a square sensor; (**b**) for a circular sensor.

Figure 15a,b include the whole set of AFM measurements carried out for the square and circular sensors experimental characterization, respectively. Both graphs present the statistical data of sensor deflection for each applied force, displaying its average value, the 25th and 75th percentiles, and the extreme collected values. With this information, a least-squares fitting procedure has been applied to obtain a linear approximation for the aforementioned data, probing the maximum deflection to be proportional to the applied force as predicted by analytical and FE simulation.

Besides probing the linearity of the sensor response under a central force, the experimental results also demonstrate that maximum deflections for both geometries are almost equal, as predicted with the analytical and FE models. Although in a different scale, both graphs in Figure 8 show that each maximum deflection value obtained with the “complex” FE model for the circular sensor matches its square counterpart. Similarly, the abovementioned linear approximation of the measurements carried out with the AFM (Figure 15) demonstrates this same behavior by showing a similar proportionality constant between maximum deflection and force for both the square and the circular plate.

**Figure 16:**
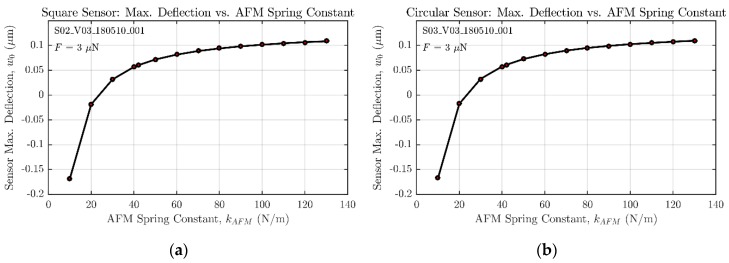
AFM cantilever spring constant influence over the estimation of sensor deflection. (**a**) Square sensor analysis; (**b**) circular sensor analysis.

However, the experimental measurements bring out a higher sensor sensitivity than expected from both FE and analytical models. This result can be explained if the impact of the spring constant value is quantified, as done in Figure 16. These two graphs show how the selection of a spring constant value for the probe significantly affects the final result for the maximum deflection of both square and circular geometries of the sensor. As an example, the tolerance range set by the manufacturer goes from 10 to 130 N/m, but values below 25 N/m would lead to unreasonable negative deflection results.

**Figure 17:**
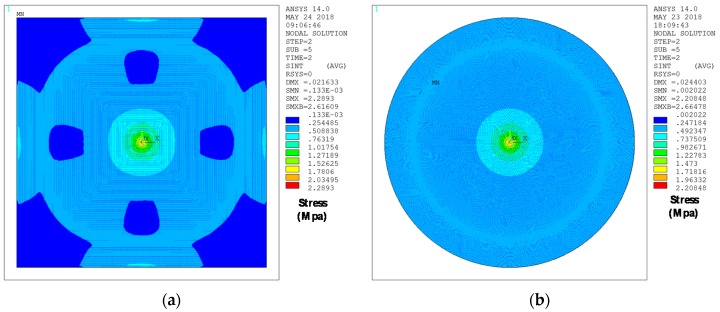
ANSYS results for the sensor stress distribution in MPa under an applied concentrated force of $F_{MAX}=3\;\mu N$ at the top plate center. (**a**) Square sensor stress distribution; (**b**) circular sensor stress distribution.

Figure 17 provides an explanation for this equal behavior in spite of considering different geometries for the sensor. In this figure, the stress distribution under the same central force for both cases is depicted. As can be seen, this force is not big enough to induce significant stress outside the immediate vicinity of the plate center, where the geometrical constraints of both types of sensors have no effect and both behave as if they were circular.

## 4. Discussion

In this work a novel characterization method for prototypes of capacitive MEMS pressure sensors using an AFM has been presented. This approach takes advantage of a relatively common laboratory instrument, applied to a non-coated prototype for biomedical applications, where sensitivity and reliability are critical. Hence, this methodology helps to reduce the cost of the testing setup while ensuring accuracy and increasing reliability. The AFM has been configured in contact mode to apply a concentrated force on the center of the top plate of the sensor, in order to obtain a force versus piezo vertical displacement from which the maximum deflection of the plate under concentrated load can be determined.

Two prototypes of MEMS sensors with different geometries (circular and square) have been submitted to AFM characterization, and the resulting experimental data have been compared with simulation results developed on both analytical and FE models. Regarding these last ones, two different FE models with variable complexity have been developed to perform deflection versus force simulations and compare the resulting bending data to the response anticipated by the analytical expressions. A first FE model, referred as “simple”, requires low computational time as it consists only of a flat square or circular plate fully clamped along its edges. The second FE model, named “complex”, presents the exact same topology as the prototype sensor fabricated in PolyMUMPS technology and it includes hole and dimple elements as required by the manufacturer.

The complete design of both prototypes of capacitive MEMS pressure sensors for ISR follow-up has been developed with a Matlab-based computer aided design (CAD) tool called CardioMEMS Design (CMD). This software tool automates the design steps, as well as provides a friendly interface to guide the user through this process that comprises three main stages: rough sensor design based on analytical expressions satisfying the initial specifications; sensor realistic 3D FE model; and sensor layout description in the corresponding technology layers to be sent to the manufacturer.

In light of the experimental results obtained by the AFM, the predicted linear behavior of the maximum deflection as a function of a concentrated load has been demonstrated. Besides that, the data extracted from AFM measurements also confirms that deflection values for both geometries of the sensor are almost equal within the applied force range. The similar stress distribution in the vicinity of the plate center that was extracted by simulation on FE models explain that geometry-independent response.

Nevertheless, the experimental measurements show a deviation in the absolute values for the maximum deflection compared with what was expected from both FE and analytical models. In this regard, the calculation method for sensor displacement is highly dependent on the spring constant, as demonstrated in the Section 3. To overcome this limitation in obtaining accurate absolute deflection values, a reference stiffness calibration method for the AFM probe could be applied.

As possible lines for future work, besides the application of the aforementioned calibration method, it will be interesting to study how this methodology can be adapted to analyze the sensor behavior against fatigue-caused aging.

## Figures and Tables

**Table 1:** Values of $A_{m}$ and $B_{m}$ parameters for a square plate [12].

m	$A_{m}$	$B_{m}$
1	−0.1025 · *p*	−0.1025 · *p*
3	0.0263 · *p*	0.0263 · *p*
5	0.0042 · *p*	0.0042 · *p*
7	0.0015 · *p*	0.0015 · *p*
9	0.00055 · *p*	0.00055 · *p*
11	0.00021 · *p*	0.00021 · *p*
13	0.00006 · *p*	0.000006 · *p*

**Table 2:** Design specifications for the square sensor’s analytical and finite element (FE) models.

Parameter	Analytic Model	FE Simple Model	FE Complex Model
Force (F)	0–3 µN	0–3 µN	0–3 µN
Top plate side ($a_{top}=b_{top}$)	410 µm	410 µm	410 µm
Bottom plate side ($a_{bottom}=b_{bottom}$)	410 µm	390 µm	390 µm
Plates gap ($t_{g}$)	2 µm	2 µm	2 µm
Top plates thickness ($t_{m}$)	2 µm	2 µm	2 µm
Number of holes	0	0	144
Number of dimples	0	0	169
Side aperture	0	0	50 µm

**Table 3:** Design specifications for the circular sensor’s analytical and FE models.

Parameter	Analytic Model	FE Simple Model	FE Complex Model
Force (F)	0–3 µN	0–3 µN	0–3 µN
Top plate radius ($a_{top}$)	220 µm	220 µm	220 µm
Bottom plate radius ($a_{bottom}$)	220 µm	210 µm	210 µm
Plates gap ($t_{g}$)	2 µm	2 µm	2 µm
Top plates thickness ($t_{m}$)	2 µm	2 µm	2 µm
Number of holes	0	0	479
Number of dimples	0	0	267
Side aperture	0	0	50 µm

**Table 4:** CardioMEMS Design (CMD)-input parameters selected to perform the sensor design.

Parameter	Value
Fabrication technology ^1^	PolyMUMPS
Diaphragm shape	Circular
Detectable pressure range	0 to 60 mmHg
Line to PAD width	40 µm

^1^ The fabrication technology must be included in the program database.
